# Causal effects of inflammatory bowel disease on brain structure: A Mendelian randomization study

**DOI:** 10.1097/MD.0000000000046867

**Published:** 2026-01-02

**Authors:** Ying He, Jian He, Chunlan Chen

**Affiliations:** a Department of Infectious Diseases, The Second Xiangya Hospital of Central South University, Clinical Medical Research Center for Viral Hepatitis in Hunan Province, Changsha, Hunan Province, China; b Department of Gastroenterology, Nanfang Hospital, Southern Medical University, Guangzhou, Guangdong Province, China; c Department of Respiratory Medicine, Hunan Provincial People’s Hospital (The First Affiliated Hospital of Hunan Normal University), Changsha, Hunan Province, China.

**Keywords:** brain structure, inflammatory bowel disease, Mendelian randomization

## Abstract

Brain structural abnormalities are common in patients with Crohn disease (CD) and ulcerative colitis (UC). However, the precise causal relationships between them remain uncertain. Hence, this study aimed to assess the causal effects of CD and UC on brain structure by using Mendelian randomization (MR) analysis. Single nucleotide polymorphisms retrieved from genome-wide association studies are usually selected as instrument variants. In this study, CD and UC-related single nucleotide polymorphisms were used as instrument variants. Two hundred brain imaging-derived phenotypes (IDPs) including regional volume, cortical area and cortical thickness were used as outcome indicators of brain structure. The inverse variance weighting as main method was performed to assess the causal effects. Additionally, potential mediators that may influence brain structure were further investigated by two-step mediation MR analysis. Among 200 IDPs, CD and UC causally influenced 30 IDPs, including regional volume (14 IDPs), cortical area (9 IDPs), and thickness (7 IDPs). In mediation MR analysis, interleukin-6 mediated the causalities from CD to the decreased volume of right frontal operculum, the shrunken area of left banks superior temporal sulcus and right caudal anterior cingulate (mediated proportion = 11.81%, 16.94%, and 11.90%, respectively). C-reactive protein mediated the causalities from CD to the shrunken area of left inferior parietal and from UC to the increased volume of right thalamus (mediated proportion = 3.92% and 5.72%, respectively). This study revealed the causal effects of CD and UC on abnormalities of specific brain regions and indicated that the causal pathways were mediated partly by interleukin-6 or C-reactive protein.

## 1. Introduction

Inflammatory bowel disease (IBD), mainly consisting of Crohn disease (CD) and ulcerative colitis (UC), is a chronic systemic inflammatory disease that affects the gastrointestinal (GI) tract and extra-GI organs.^[1]^ Patients with IBD are usually diagnosed in early adulthood and have lifelong symptoms due to chronic systemic inflammation.^[2]^ The interaction of genetic susceptibility, gut microbiota, and environmental risk factors is key in the development of IBD,^[3,4]^ which could affect multiple organs, for example the skin, liver, eyes, and brain. Brain structural abnormalities are frequently observed in IBD patients, which may be a crucial factor in the development of neuropsychiatric disorders in these patients. Growing evidence has demonstrated the causal effects of brain structural abnormalities on neuropsychiatric disorders^[5–8]^; however, the impact of IBD on brain structure remains unknown. The production of a large amount of inflammatory factors is the main pathophysiological characteristic of patients with IBD. Intestinal inflammatory signals may be transmitted to the brain through the gut–brain axis, altering the structure and function of brain.^[9]^ A causal association might exist between inflammation and brain structures. Such as, interleukin-6 (IL-6) is one of the most important inflammatory biomarker and plays a vital role in the development of IBD.^[10]^ The increase of serum IL-6 level could result in changes in brain structure and lead to neuropsychiatric disorders.^[11–13]^ In addition, high C-reactive protein (CRP) level is associated with regional volume abnormalities, Alzheimer disease, and depression. Therefore, inflammatory factors are not only closely related to the development of IBD, but also affects the structure and function of brain through the gut–brain axis.

Neuropsychiatric disorders including depression, anxiety, schizophrenia, and neurodegenerative diseases are common in IBD patients and bring huge psychological burdens to families.^[14–16]^ The pooled incidence of anxiety and depression symptoms in IBD patients was 32.1% and 25.2%, which was significantly higher than general population (3.4%).^[17–19]^ Several population-based cohort studies found the significant changes in the brain gray and white matter in patients with CD or UC,^[20,21]^ which may account for the higher prevalence of anxiety and depression in those patients. Additionally, the reduced gray matter volume of right superior temporal gyrus is closely related to the occurrence of schizophrenia.^[22]^ The incidence of IBD among patients with schizophrenia was significantly higher than matched controls (1.14% vs 0.25%).^[23]^ Clarifying the pathogenic underpinning of neuropsychiatric disorders is of great clinical significance for the management of IBD patients.

The brain structural abnormalities commonly found in patients with IBD imply a potential causal relationship between IBD and brain structure.^[20,21,24]^ However, conventional observational studies have reverse causality and cannot control for effects of confounders. In addition, in the real world, it is difficult to elucidate the impact of IBD on brain structure via randomized controlled trials. Hence, the precise causal effect of IBD on brain structure is unclear. To address this gap, Mendelian randomization (MR), which is analogous to randomized controlled trials, emerges as a widely used, robust, and high quality epidemiological method.^[25,26]^ In MR analysis, genetic instrumental variables (IVs) are adopted to assess the causal effect of the exposure on the outcome. Single nucleotide polymorphisms (SNPs) retrieved from large-scale open-access genome-wide association studies (GWAS) are usually selected as IVs.^[27]^

Thus, this study used MR analysis to evaluate the causal effects of CD and UC on brain structure. CD- and UC-associated SNPs from the GWAS summary statistics were used as genetic IVs. Two hundred brain imaging-derived phenotypes (IDPs) including regional volume, cortical area, and cortical thickness, measured by noninvasively magnetic resonance imaging (MRI), were used as outcome indicators of brain structure.^[6,7,28,29]^ In addition, we conducted a two-step mediation MR analysis to assess the mediating effects of inflammatory cytokines on the causal pathways from CD and UC to brain structural abnormalities.

## 2. Methods

### 2.1. GWAS summary statistics source for CD, UC, and brain IDPs

The GWAS summary statistics for CD (17,897 patients, 33,977 controls) and UC (13,768 patients, 33,977 controls),^[30]^ individuals of European descent, were from the International Inflammatory Bowel Disease Genetics Consortium (IIBDGC) to obtain more comprehensive and reliable results (Table S1, Supplemental Digital Content, https://links.lww.com/MD/R65). UC and CD were diagnosed by physicians based on the comprehensive evidence of clinical symptoms, endoscopic findings, and histopathological and imaging results. The GWAS summary statistics were from 7 CD and 8 UC cohorts with combined genome-wide SNP data. The proportion of males in the CD and UC cohorts was 45% and 52%, respectively. The Affymetrix Genome-Wide Human SNP Array 6.0, Illumina HumanHap300 BeadChip and Illumina HumanHap550 BeadChip arrays were used in combination to genotype the samples.^[30]^ IIBDGC is an authoritative organization devoted to identification of genetic risk factors for IBD; the Consortium is composed of a global network of hundreds of researchers from >20 countries and collects GWAS data from over 75,000 patients with IBD. The independent CD- and UC-related SNPs are listed in Tables S2 and S3, Supplemental Digital Content, https://links.lww.com/MD/R65, respectively.

For brain IDPs GWAS summary statistics, the brain imaging data were processed by the Wellcome Centre for Integrative Neuroimaging on behalf of UK Biobank (UKB). IDPs GWAS summary statistics were freely downloaded at https://open.win.ox.ac.uk/ukbiobank/big40/ and used as the outcomes.^[6,31]^ Brain structure and function can be measured by using noninvasively MRI.^[32]^ The UKB brain imaging protocol includes 6 different modalities comprising structural, diffusion and functional imaging. The original data from these 6 modalities have been processed to generate a series of IDPs.^[33,34]^ The FreeSurfer v6.0.0 based on the Desikan-Killiany atlas were used to model cortical measurements.^[35]^ The FIRST tool and the FreeSurfer v6.0.0 based on the automatic subcortical segmentation were used to model subcortical measurements.^[36]^ Brain IDPs mostly have traceable genetic information and are functionally related to brain structures.^[31]^ The imaging manifestations of neuropsychiatric disorders such as depression, anxiety, and schizophrenia can be found in brain MRI data. Therefore, brain IDPs generated by MRI provide intermediate endophenotypes that can be used to evaluate the genetic architecture of such disorders.^[32]^ We selected 200 IDPs, including 64 IDPs of regional and tissue volume, 68 IDPs of the cortical area, and 68 IDPs of cortical thickness (Table S4, Supplemental Digital Content, https://links.lww.com/MD/R65). The sample size ranges from 31,966 to 33,224 individuals of European descent, and the proportion of males is 48%.^[31]^

The GWAS summary statistics for CD/UC and brain IDPs were from different cohorts; thus, there was no potential genetic overlap between CD/UC and brain IDPs. We used published GWAS summary statistics and did not collect original data. Participant informed consent and ethics approval had already been obtained in the preliminary studies. Therefore, these ethics materials were not required for this study. We thank the ethical considerations that were addressed in the original studies by Liu et al^[30]^ and Smith et al.^[31]^

### 2.2. Genetic IVs selection

**Figure 1.**
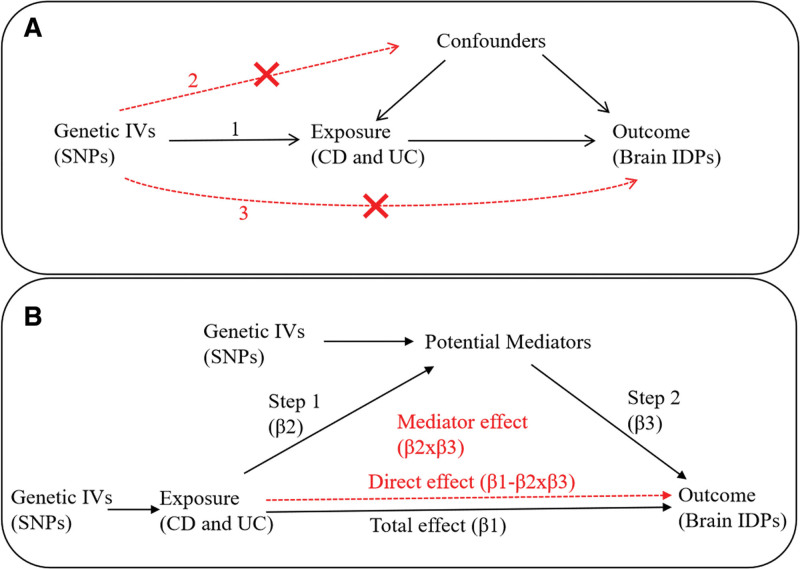
Schematic overview of the MR study and mediation analysis. (A) There were 3 principal assumptions in MR analysis: (1) relevance assumption: the genetic IVs are related to the exposure; (2) independence assumption: the genetic IVs do not affect the outcome through the confounders; (3) exclusion assumption: the genetic IVs do not affect the outcome directly, but only via indirect exposure. (B) Mediator effect of potential mediators on the causal pathway from CD and UC to brain IDPs. Total effect means the IVW-derived effect size from CD and UC to brain IDPs (β1); mediator effect means the IVW-derived effect size from CD and UC to brain IDPs via potential mediators (β2×β3); direct effect means the IVW-derived effect size from CD and UC to brain IDPs excluding mediator effect (β1‐β2×β3). CD = Crohn disease; IDPs = imaging-derived phenotypes; IVs = instrumental variables; MR = Mendelian randomization; SNPs = single nucleotide polymorphisms; UC = ulcerative colitis.

To select eligible SNPs as IVs from the GWAS summary statistics of CD and UC, the 3 following assumptions must have been satisfied^[37,38]^: relevance assumption: the IV is strongly related to the exposure; independence assumption: the IV does not share common causes with the outcome; exclusion assumption: the IV affects the outcome only by the exposure (Fig. 1A).

To satisfy the relevance assumption, the following conditions were set to select the exposure-related SNPs: strong genome-wide significant correlation with the CD or UC (*F* > 10, *P* < 5 × 10^‐8^). The *P*-value <5 × 10^‐8^ was almost used in MR study to screen that the SNP has genome-wide significant association with exposure. We used the *F* value to assess the association of instrument strength with CD or UC. When the *F* value is <10, the SNP is usually considered to be a weak IV, which may have some bias on the analysis results. The *F* value for a single SNP should be over 10 to avoid weak instrument bias and was calculated by the following equation^[37]^: *F* = [β/se]^2^, β means estimated effect size and SE means standard error of β; independent SNPs are identified by linkage disequilibrium clumping (within a 1 Mb window size, *r*^2^ < 0.001).^[37]^ To satisfy the independence assumption and exclusion assumption, we checked each SNP associated with CD and UC using PhenoScanner (http://www.phenoscanner.medschl.cam.ac.uk/) and eliminated SNPs significantly related to the potential confounding factors (smoking, body mass index, hyperlipemia, hypertension)^[28]^ and outcome. When identifying the SNPs associated to the potential confounders using PhenoScanner, the *P*-value threshold is 1E-5, *r*^2^ is 0.8 and genomic build is 37. The harmonization process was used to unify the effect allele and effect direction, ensure SNP with a minor allele frequency of >0.01, and remove the palindromic and incompatible SNPs. The incompatible SNPs were considered to be those in which the effect allele or non-effect allele in the GWAS of the exposure is not identical to the effect allele or non-effect allele in the GWAS of the outcome. For example, the effect allele and non-effect allele in the exposure are A/G, whereas they are A/C in the outcome.

### 2.3. Mendelian randomization analysis

**Figure 2.**
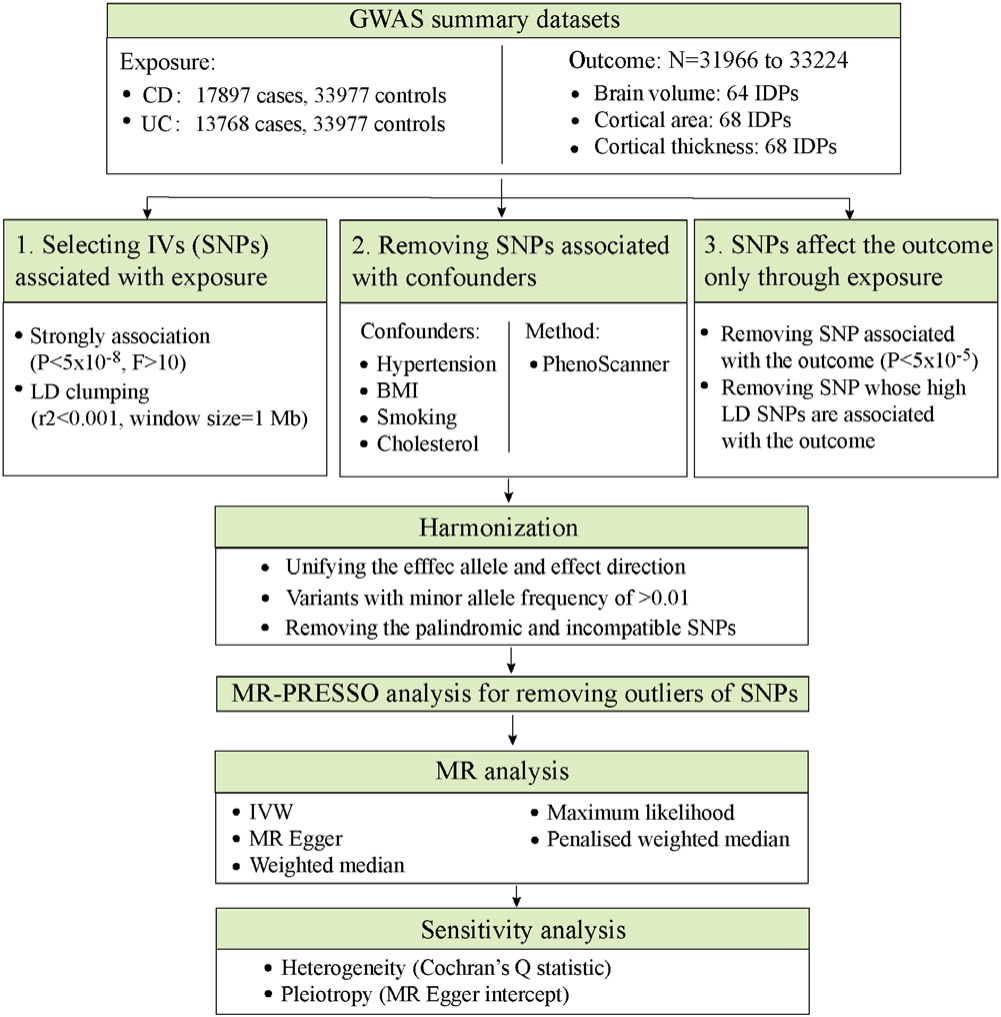
Workflow of Mendelian randomization study. BMI = body mass index; CD = Crohn disease; IDPs = imaging-derived phenotypes; IVs = instrumental variables; IVW = inverse variance weighted; LD = linkage disequilibrium; SNPs = single nucleotide polymorphisms; UC = ulcerative colitis.

First, the above-mentioned 3 assumptions were used to identify the confounder-independent SNPs as genetic IVs associated with CD and UC. Second, after the harmonization, the Mendelian randomization-pleiotropy residual sum and outlier (MR-PRESSO) test was applied to remove the outlier SNPs. Third, after removal of the outlier SNPs, the MR analysis was conducted, and the sensitivity analysis was conducted to decide whether pleiotropy existed and which inverse-variance weighted (IVW) model should be adopted according to the heterogeneity. Figure 2 shows the MR analysis procedure.

We used 5 different methods in our MR analysis, namely, IVW, MR Egger, weighted median, maximum likelihood, and penalized weighted median. The IVW, a weighted mean of individual variant effects on the outcome, was used as the main method, which provides an estimate equivalent to MR using individual-level data.^[39]^ If the sample size was large enough and the pleiotropy of IVs was not significant, the IVW-derived estimation was efficient and close to the true value.^[40]^ If significant heterogeneity (IVW Cochran *Q* statistic *P* < .05) was observed, the random effect IVW model was used; otherwise, the fixed effects IVW model was used.^[41]^ Based on the assumption that all genetic instruments are valid, the IVW method has optimal statistical power; however, its results may be biased in the presence of horizontal pleiotropy of any SNPs.^[42]^ Therefore, we performed additional 4 MR methods to address variant heterogeneity and the pleiotropy effect, which will enhance the reliability of results. The MR-Egger method, a weighted mode-based estimation method that provides an unbiased estimate even if all SNPs have pleiotropic effects, but requires that the pleiotropic effects are independent of the variant-exposure association.^[42]^ Weighted median can provide unbiased estimate when up to 50% of invalid IVs, but may be less efficient.^[42]^ Maximum likelihood assumes a bivariate normal distribution for the associations of each genetic variant with the exposure and outcome.^[43]^ Penalized weighted median is equivalent to weighted median when there is no heterogeneity, and downweights the contribution of heterogeneous variants if there is directional pleiotropy.^[40]^ Although these other methods have less statistical power (wider confidence interval), they can provide more robust and reliable causal estimations across a wider range of scenarios.^[44]^

The MR-PRESSO method was used to detect the outlier SNPs in MR analysis.^[45]^ Eligible IVs must satisfy the exclusion assumption; however, if the association of genetic IV with the outcome is independent of the exposure rather than through other traits, it is called as horizontal pleiotropy. If horizontal pleiotropy is present, the causal assessment of the exposure on outcome cannot be supported. The MR-PRESSO test detects and corrects the horizontal pleiotropy by removing the outlier SNPs one by one. More than 50% of the SNPs with balanced pleiotropy were required in the MR-PRESSO test.^[45]^ The number of distributions was set to 3000 in the MR-PRESSO test.

An adjusted *P*-value <.00025 (0.05/200, 200 denotes the number of independent hypotheses) after Bonferroni multiple testing correction was considered as statistically significant, meanwhile *P*-value between .00025 and 0.05 was considered as nominally significant. All statistical analyses were conducted using R (version 4.2.1) and the Two-Sample MR package (version 0.5.6).

### 2.4. Sensitivity analysis

The MR Egger method was used to detect the potential pleiotropy of SNPs. If the MR Egger-derived intercept *P*-value was >.05, it would be considered as no pleiotropy.^[37]^ If pleiotropy is present, the causal assessment of the exposure on outcome cannot be supported and the IVW-derived results are considered as unreliable. The heterogeneity was detected by IVW-derived Cochran *Q* statistic. If the Cochran *Q P*-value was >.05, it would be considered as no heterogeneity.

### 2.5. Mediation analysis

For significant causal associations of CD/UC with brain structure, we choose several inflammatory regulators as potential mediators to perform a two-step MR analysis. The GWAS summary statistics for IL-1α, IL-1β, IL-12, and IL-23, from genomic atlas of 3622 human plasma proteins, were obtained from the INTERVAL study, spanning 2 nonoverlapping cohorts and comprising 3301 participants of European ancestry.^[46]^ The GWAS summary statistics for IL-2, IL-10, IL-17, and TNF-α, from pharmaceutical target evaluation of 48 cytokines in inflammatory disease, were extracted from a population-based cross-sectional study spanning 3 cohorts in Finland and comprising 3475, 7681, 7760, and 3454 of European ancestry, respectively.^[47]^ The GWAS summary statistics for IL-6, IL-27, and MCP-1, from the genomic and drug target evaluation of 90 cardiovascular proteins, were extracted from a comprehensive analysis spanning 13 cohorts and comprising 21,758 individuals of European ancestry.^[48]^ The GWAS summary statistics for CRP, from the analysis of genetic determinants for chronic inflammation, were extracted from a meta-analysis including 88 studies and 204,402 participants of European ancestry.^[49]^

In the first step, CD- and UC-associated SNPs as IVs were used to test the causal effects of CD and UC on 12 potential mediators. An adjusted *P*-value < .0041 (0.05/12, 12 denotes the number of independent hypotheses) after Bonferroni multiple testing correction was considered as statistically significant, meanwhile *P*-value between .0041 and .05 was considered as nominally significant. In the second step, identified mediator-associated SNPs as IVs were used to assess the causality of mediators on brain IDPs (Fig. 1B).

The MR analysis methods and sensitivity analysis were similar to the above-mentioned methods. The total effect, mediator effect, direct effect, and mediated proportion were calculated to assess the role of mediator in the causal pathways.^[50,51]^ Total effect refers to the IVW-derived effect size from the exposure to the outcome (β1). Mediator effect is the IVW-derived effect size from the exposure to the outcome via potential mediators (β2×β3). Direct effect is the IVW-derived effect size from the exposure to the outcome excluding mediator effect (β1‐β2×β3). We applied delta method with RMediation package to calculate mediation effect and 95% confidence interval. The mediated proportion was used to estimate the extent of the mediating role in the causal effect of the exposure on the outcomes and was calculated by the equation mediated proportion = [(β2×β3)/β1] × 100%.

## 3. Results

### 3.1. Description of selecting IVs

After the linkage disequilibrium clumping and harmonization, the outlier SNPs were removed by using the MR-PRESSO test. Since we used 200 brain IDPs from distinct GWAS summary statistics as outcomes, the numbers of exposure-related SNPs varied for each IDP after harmonization process and MR-PRESSO test. Finally, 99 to 102 independently CD-related SNPs and 71 to 73 independently UC-related SNPs were identified for estimating the causal effects on 200 brain IDPs. Detailed SNPs of CD and UC are provided in Tables S2 and S3, Supplemental Digital Content, https://links.lww.com/MD/R65.

### 3.2. Causal estimates of CD and UC on brain structure

**Figure 3.**
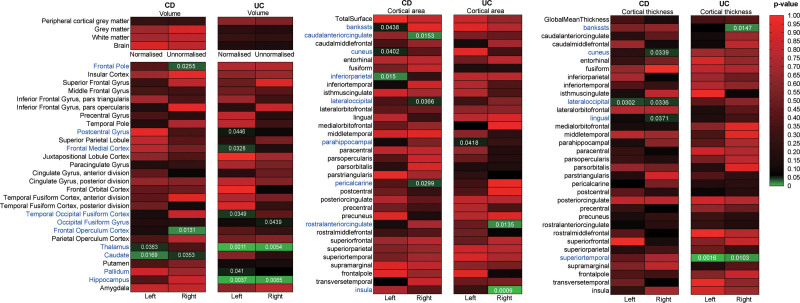
Heat maps showing IVW estimates of CD and UC on brain IDPs. The color of each block represents the IVW-derived *P*-value. The *P*-values of <.05 were shown in green, otherwise shown in black or red. The Bonferroni-corrected *P*-value < .00025 is considered as statistically significant, whereas *P*-value < .05 is considered as nominally significant. CD = Crohn disease; IVW = inverse variance weighted; UC = ulcerative colitis.

The IVW-derived *P*-values for the causal estimates from CD and UC to 200 brain IDPs are presented in the heat maps of Figure 3, and the causal estimates from other MR methods are shown in Tables S5 and S6, Supplemental Digital Content, https://links.lww.com/MD/R65.

Among 200 brain IDPs, CD was identified to have significant causal effects on 15 brain IDPs including regional volume (5 IDPs), cortical surface (6 IDPs) and thickness (4 IDPs) (Table 1 and Tables S7–S9, Supplemental Digital Content, https://links.lww.com/MD/R65). For regional volume, the genetic predisposition for CD was related to the decreased volume of right frontal pole (β = ‐0.0149) and right frontal operculum (β = ‐0.0166) and associated with the increased volume of left thalamus (β = 0.0172) and both sides of caudate (β^left^ = 0.0184, β^right^ = 0.0165). For cortical area, CD significantly affected the enlargement of left cuneus (β = 0.0172), right lateral occipital (β = 0.0143), and right pericalcarine (β = 0.0148), and affected the decreased area of left banks superior temporal sulcus (β = ‐0.0137), left inferior parietal (β = ‐0.0166), and right caudal anterior cingulate (β = ‐0.0165). For cortical thickness, CD has significantly causal effects on the reduction of right cuneus (β = ‐0.0174), lingual gyrus (β = ‐0.0143), and both sides of lateral occipital (β^left^ = ‐0.018, β^right^ = ‐0.0172).

**Table 1:** Significant MR estimates by IVW method from CD to regional volume, cortical area, and cortical thickness.

Exposure (CD)	Outcome (IDP ID)	SNPs (N)	β (95% CI) IVW	*P*-value (IVW)	Cochran *Q P*-value (IVW)	Intercept *P*-value (MR-Egger)
Volume	Right frontal pole (0027)	102	‐0.0149 (‐0.028; ‐0.002)	.0255	0.0703	0.7759
	Right frontal operculum cortex (0107)	102	‐0.0166 (‐0.03; ‐0.003)	.0131	0.2096	0.1317
	Left thalamus (0122)	101	0.0172 (0.001; 0.033)	.0363	0.0008	0.8805
	Left caudate (0124)	100	0.0184 (0.003; 0.033)	.0169	0.0206	0.4556
	Right caudate (0125)	100	0.0165 (0.001; 0.017)	.0353	0.0107	0.1512
Cortical area	Left bankssts (0649)	102	‐0.0137 (‐0.027; ‐0.0003)	.0438	0.0617	0.5652
	Left cuneus (0652)	101	0.0172 (0.001; 0.033)	.0402	0.0008	0.2011
	Left inferior parietal (0655)	102	‐0.0166 (‐0.03; ‐0.003)	.015	0.2635	0.8728
	Right caudal anterior cingulate (0684)	102	‐0.0165 (‐0.03; ‐0.003)	.0153	0.0733	0.7235
	Right lateral occipital (0692)	101	0.0143 (0.001; 0.028)	.0366	0.1215	0.4307
	Right pericalcarine (0702)	101	0.0148 (0.001; 0.028)	.0299	0.4107	0.5349
Cortical thickness	Left lateral occipital (1030)	101	‐0.018 (‐0.034; ‐0.002)	.0302	0.0015	0.8727
	Right cuneus (1058)	101	‐0.0174 (‐0.033; ‐0.001)	.0339	0.0028	0.2756
	Right lateral occipital (1064)	100	‐0.0172 (‐0.033; ‐0.001)	.0336	0.0055	0.286
	Right lingual gyrus (1066)	101	‐0.0143 (‐0.014; ‐0.001)	.0371	0.1923	0.3177

CD = Crohn disease, CI = confidence interval, IDP = imaging-derived phenotypes, IVW = inverse-variance weighted, MR = Mendelian randomization, SNP = single nucleotide polymorphisms.

From UC to 200 brain IDPs, our results showed that UC significantly affected 15 brain IDPs, including volume (9 IDPs), cortical surface (3 IDPs), and thickness (3 IDPs) (Table 2). For regional volume, the genetic predisposition for UC was related to the risk of increased volume of left postcentral gyrus (β = 0.0165), frontal medial (β = 0.0176), temporal occipital fusiform (β = 0.0174), right occipital fusiform gyrus (β = 0.0166), both sides of thalamus (β^left^ = 0.0268, β^right^ = 0.0229), and hippocampus (β^left^ = 0.0224, β^right^ = 0.027). In addition, UC was related to the decreased volume of left pallidum (β = ‐0.0191). For the cortical area, UC significantly affected the enlarged area of left para-hippocampal gyrus (β = 0.0171), right rostral anterior cingulate (β = 0.0208), and, instead, the reduced right insula area (β = ‐0.0281). For cortical thickness, UC significantly affected the increased thickness of left superior temporal gyrus (β = 0.0203), right banks superior temporal sulcus (β = 0.0205), and superior temporal gyrus (β = 0.0215).

**Table 2:** Significant MR estimates by IVW method from UC to regional volume, cortical area, and cortical thickness.

Exposure (UC)	Outcome (IDP ID)	SNPs (N)	β (95% CI) IVW	*P*-value (IVW)	Cochran *Q P*-value (IVW)	Intercept *P*-value (MR-Egger)
Volume	Left postcentral gyrus (0058)	73	0.0165 (0.0004; 0.0325)	.0446	0.0705	0.1355
	Left frontal medial cortex (0074)	73	0.0176 (0.0015; 0.0336)	.0328	0.3296	0.1656
	Left temporal occipital fusiform cortex (0102)	73	0.0174 (0.0013; 0.0334)	.0349	0.5012	0.1097
	Right occipital fusiform gyrus (0105)	73	0.0166 (0.0005; 0.0326)	.0439	0.092	0.3927
	Left thalamus (0122)	73	0.0268 (0.0107: 0.0428)	.0011	0.1673	0.1169
	Right thalamus (0123)	73	0.0229 (0.0068; 0.0389)	.0054	0.0911	0.5075
	Left pallidum (0128)	73	‐0.0191 (‐0.0381; ‐0.0001)	.041	0.0168	0.508
	Left hippocampus (0130)	72	0.0224 (0.0077; 0.0402)	.0037	0.0765	0.9302
	Right hippocampus (0131)	73	0.027 (0.0076; 0.0464)	.0065	0.0072	0.1271
Cortical area	Left parahippocampal gyrus (0663)	73	0.0171 (0.0064; 0.0335)	.0418	0.1995	0.9606
	Right rostral anterior cingulate cortex (0707)	73	0.0208 (0.0043; 0.0372)	.0135	0.6114	0.2009
	Right insula (0715)	72	‐0.0281 (‐0.0445; ‐0.0116)	.0009	0.3119	0.151
Cortical thickness	Left superior temporal gyrus (1049)	72	0.0203 (0.0036; 0.0369)	.0016	0.1519	0.4667
	Right banks superior temporal sulcus (1055)	73	0.0205 (0.004; 0.0369)	.0147	0.5156	0.3372
	Right superior temporal gyrus (1083)	73	0.0215 (0.0048; 0.0381)	.0103	0.1878	0.8701

CI = confidence interval, IDP = imaging-derived phenotypes, IVW = inverse-variance weighted, MR = Mendelian randomization, SNP = single nucleotide polymorphisms, UC = ulcerative colitis.

Additionally, the estimations of another 4 supplementary MR analyses were in the same direction as IVW, which indicated the robustness of causality (Tables S5 and S6, Supplemental Digital Content, https://links.lww.com/MD/R65). Even though the IVW-derived *P*-values of above mentioned results were between 0.00025 and 0.05, it was important to point out that these results indicated nominally causal associations, due to the use of strict Bonferroni multiple correction in this study. Taken together, these results showed that the genetic susceptibility to CD and UC was associated with the risk of abnormalities of specific brain regions involved in regional volume, cortical area and thickness.

### 3.3. Mediation estimates for the causal pathways from CD and UC to brain structure

CD and UC are chronic inflammatory diseases, so their effects on brain structure may be mediated by inflammatory factors that function by way of the gut–brain axis. Therefore, we next assessed the mediation effects of 12 cytokines (IL-1α, IL-1β, IL-2, IL-6, IL-10, IL-12, IL-17, IL-23, IL-27, TNF-α, CRP, and MCP-1) on causal pathways from CD and UC to brain structure by two-step mediation MR analysis. Detailed information for the GWAS summary statistics of the inflammatory cytokines is shown in Table S10, Supplemental Digital Content, https://links.lww.com/MD/R65.

In step 1, CD- or UC-associated SNPs were used to assess the causal associations with inflammatory factors. The genetic predisposition for CD was associated with increased levels of IL-6 (β = 0.0275, *P* = .004), IL-27 (β = 0.0234, *P* = .0144), and CRP (β = 0.0124, *P* = .0342). UC was associated with increased levels of IL-6 (β = 0.0244, *P* = .041) and CRP (β = 0.0218, *P* = .0084) (Table S11, Supplemental Digital Content, https://links.lww.com/MD/R65). Notably, only the IVW-derived *P*-values of IL-6 <.0041 after Bonferroni multiple correction was considered as statistically significant, while others were considered as nominally significant. Supplementary MR analysis and MR-Egger intercept *P*-value verified the reliability of the results. In step 2, SNPs associated with IL-6, IL-27, or CRP were used to assess the causal associations with the 30 brain IDPs causally affected by CD and UC. Detailed information of independent SNPs for CRP, IL-6, and IL-27 is shown in Table S12, Supplemental Digital Content, https://links.lww.com/MD/R65. IL-6, IL-27, and CRP were identified to significantly affect genetically 7, 2, and 1 brain IDPs, respectively (Table S13, Supplemental Digital Content, https://links.lww.com/MD/R65).

**Figure 4.**
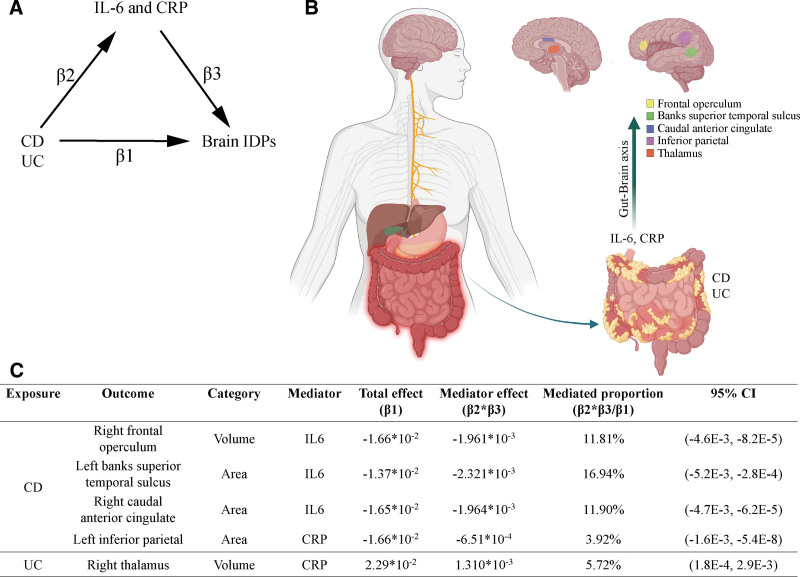
Illustration and results of mediation analysis. (A) Directed acyclic graph shows the mediation Mendelian randomization analysis to assess the mediating effects of IL-6 and CRP on the causal pathway from CD and UC to brain structure. β1 means the IVW-derived effect size from CD and UC to brain IDPs; β2 means the IVW-derived effect size from CD and UC to mediators (IL-6 and CRP); β3 means the IVW-derived effect size from mediators (IL-6 and CRP) to brain IDPs. (B) Schematic shows that the specific brain anatomical regions causally affected by CD and UC were mediated by IL-6 and CRP, probably by way of the gut–brain axis. (C) Significant two-step mediation MR analysis by IVW method for mediation effects of IL-6 and CRP on the causal pathway from CD and UC to brain structure. CD = Crohn disease; CRP = C-reactive protein; IDPs = imaging-derived phenotypes; IL-6 = interleukin-6; IVW = inverse variance weighted; UC = ulcerative colitis.

For mediation estimates of the causal effects, the orientation of the total effect (β1) must be consistent with the orientation of the mediator effect (β2×β3) (Fig. 4A). Hence, we identified only 5 mediating MR networks (Fig. 4B and Tables S14 and S15, Supplemental Digital Content, https://links.lww.com/MD/R65). Causal effects of CD on the decreased volume of right frontal operculum (mediator proportion = 11.81%), shrunken area of left banks superior temporal sulcus (mediator proportion = 16.94%) and right caudal anterior cingulate (mediator proportion = 11.90%) were mediated by IL-6, whereas the shrunken area of left inferior parietal (mediator proportion = 3.92%) was mediated by CRP (Fig. 4C). In addition, elevated genetic UC predisposition enlarged the volume of right thalamus via CRP (mediator proportion = 5.72%) (Fig. 4C). IL-6 and CRP, as important inflammatory markers in IBD and neuropsychiatric disorders, may be crucial mediators in gut–brain axis affecting brain structure and function. Taken together, our results suggested that the causal pathways from CD and UC to 5 brain IDPs were mediated partly by IL-6 and CRP.

## 4. Discussion

Clinical observational studies have revealed brain structural abnormalities in both patients with CD and UC patients; however, whether this relation is causal was unknown. Our study is the first to systematically investigate the potential causal effects of CD and UC on brain structure by two-sample MR analysis based on the GWAS summary statistics from large-scale European ancestry populations. We found that CD and UC have causal effects on 30 brain IDPs, including the regional volume (14 IDPs), cortical area (9 IDPs), and cortical thickness (7 IDPs), with varying degree in the form of enlargement or decrease. On the basis of the existence of the gut–brain axis between the GI tract and central nervous system,^[52,53]^ we sought to decipher the complex causal pathways from CD and UC to brain structural abnormalities from the perspective of inflammation. Among the 30 IDPs, we revealed that the causal pathways from CD or UC to regional volume (frontal operculum and thalamus) and cortical area (banks superior temporal sulcus, inferior parietal and caudal anterior cingulate) were mediated partly by genetically predicted IL-6 and CRP.

The caudate has a critical function in high-level neurological processes, such as learning, memory, motivation, and emotion.^[54]^ Dysfunction of the caudate contributes to dementia, schizophrenia, Parkinson disease, and autism.^[55]^ The pallidum is involved in the regulation of voluntary movement.^[56]^ The hippocampus functions in response inhibition, episodic memory, and spatial cognition.^[57]^ Damage to the hippocampus is closely associated with neuropsychiatric disorders such as Alzheimer disease and schizophrenia.^[58,59]^ We found that CD causally affects the increased volume of thalamus and caudate, and UC causally affects the increased volume of thalamus and hippocampus and the decreased volume of pallidum. Our findings are consistent with the previous observational studies of patients with IBD.^[20,21]^ In addition, the cortical area and thickness of some regions were affected to varying degree by CD and UC. Our study suggests that attention should be focused on brain magnetic resonance imaging examination of patients with IBD, especially patients with neuropsychiatric symptoms, because such patients with IBD may have brain structural abnormalities. A comprehensive brain MRI examination would be beneficial for treatment and prognosis assessment of patients with IBD.

The gut–brain axis influenced by dysregulated inflammation can affect neuronal development and subsequent behavioral phenotypes, even leading to neuropsychiatric disorders. We identified CRP and IL-6 as genetically predicted inflammatory mediators in the causal pathways from CD and UC to 5 brain IDPs. Although we tested 12 inflammatory cytokines, CD and UC causally affected only the increased levels of CRP and IL-6. The increased volume of thalamus in UC was mediated by increased CRP, which agreed with the finding that the volume of the thalamus was positively correlated with CRP concentration.^[24]^ In addition, the decreased cortical area of the left inferior parietal in CD was mediated by CRP, which agreed with a report that the volume in the inferior parietal was decreased in patients with CD.^[20]^ In addition, the causal effects of CD on the decreased volume of right frontal operculum cortex, left banks superior temporal sulcus, and right caudal anterior cingulate were mediated by IL-6 instead of CRP.

IL-6 and CRP are 2 functionally related inflammatory biomarkers. At the acute phase of inflammatory response, the induction of CRP in the liver is mainly dependent on the stimulation by IL-6. With the increase of IL-6, the concentration of CRP is significantly increased, rising from 1 μg/mL to 1000 μg/mL during acute phase of inflammation in severe systemic infection. The CRP in plasma begin to increase 4 to 6 hours after the onset of infection, and reached the peak at about 24 to 48 hours. Additionally, in many cases, chronic inflammation is derived from the moderate elevations of IL-6 and CRP. Large-scale cohort studies and meta-analysis provide the strong evidence that the high levels of IL-6 and CRP are closely related to the poor outcomes due to various causes, including cancer, cardiovascular disease and the metabolic syndrome.^[60]^ High CRP level is associated with regional volume abnormalities,^[61–63]^ Alzheimer disease,^[64,65]^ and depression.^[66,67]^ IL-6 exerts important functions in integrating behavioral phenotypes, neurobiology, and immunology. High IL-6 level is associated with neuronal development,^[13]^ brain repair,^[11]^ and multiple neuropsychiatric disorders.^[7,12]^ Recent findings have shown that CRP exerts risk-increasing effects on anorexia nervosa, major depressive disorder, and obsessive-compulsive disorder, while it displays protective effect on schizophrenia and Alzheimer disease.^[68,69]^ An another study showed strong evidence that increased CRP level in patients with advanced liver fibrosis significantly mediated the effect of liver fibrosis on cognitive functioning and regional gray matter volumes in the hippocampus and brain stem.^[70]^ Although epidemiological studies provide evidence for the strong associations of IL-6 and CRP with structural abnormalities and dysfunction of the brain, the signaling pathways and mechanisms are not yet clear. A plausible explanation is that IL-6 and its receptor can cross the blood-brain barrier and increase its permeability, thereby aggravating local inflammation, which could be related to treatment resistance and impaired brain function.^[71,72]^ In addition, neuronal pathway mediated by gut afferent vagal neurons is important for the gut–brain axis. Stimulation of intestinal vagus afferent fibers by inflammatory cytokines through vagal receptors triggers the hypothalamic–pituitary–adrenal axis and activates neural circuits related to disease behavior. Hence, IL-6 may activate intestinal vagal afferents to relay signals to specific brain regions, and then affect the brain function.

The strengths of our study include the following aspects. The large-scale GWAS summary-level data of exposure and outcome were both obtained from European individuals, which avoided the bias caused by different ethnic groups. We used 200 brain IDPs covering most of the brain regions as outcomes and provided a large number of reliable results with potential clinical implications. By conducting a two-step MR analysis, we found that IL-6 and CRP were mediators of the causal pathways from CD and UC to brain structure.

In addition, we note several limitations in this study. The patients diagnosed with CD or UC were from different medical units, and the differences in diagnostic approach, data collection, and data processing may bring bias to the results and contribute to heterogeneity. Both UKB and IIBDGC participants were Europeans; thus, the generalizability of the results to other ethnic groups was limited. A large-scale cross-ancestries cohort study is required to clarify the associations between IBD and brain structure. CD and UC have diverse severity, status, age, and drug treatments; however, stratification analyses were not possible because we used summary statistics, which may bias the results. The small IVs sample sizes may result in false negative results, undermine the reliability, limit statistical power, etc. Because low amounts of IVs do not adequately capture the variation in exposure, causal estimates may be biased. Therefore, the causal estimates with small IVs sample sizes should be interpreted with caution.

## 5. Conclusions

This MR analysis revealed the causal effects of CD and UC on brain structural abnormalities and indicated that the causal pathways were mediated partly by IL-6 and CRP, which endorsed the effects of chronic inflammation on brain structure and function. The findings will deepen the understanding of the pathogenesis of neuropsychiatric disorders in IBD and the function of gut-brain axis regulated by inflammation, and may provide new ideas for the treatment strategies of IBD.

## Acknowledgments

The authors thank all the investigators and participants of the IIBDGC, UK Biobank, Centre for Integrative Neuroimaging, and IEU OpenGWAS project.

## Author contributions

**Conceptualization:** Ying He.

**Data curation:** Ying He.

**Formal analysis:** Jian He.

**Funding acquisition:** Ying He, Chunlan Chen.

**Investigation:** Ying He.

**Methodology:** Ying He.

**Project administration:** Chunlan Chen.

**Resources:** Jian He.

**Software:** Ying He.

**Supervision:** Chunlan Chen.

**Validation:** Jian He.

**Visualization:** Ying He, Jian He.

**Writing – original draft:** Ying He.

**Writing – review & editing:** Chunlan Chen.

## Supplementary Material


